# A unique case of AH-dominant type nodular pulmonary amyloidosis presenting as a spontaneous pneumothorax: a case report and review of the literature

**DOI:** 10.3389/pore.2023.1611390

**Published:** 2023-09-22

**Authors:** Valeria Skopelidou, Pavel Hurník, Lubomír Tulinský, Vladimir Židlík, Jiří Lenz, Patricie Delongová, Helena Hornychová, Patrik Flodr, Tomáš Jelínek, Ludmila Muroňová, Dušan Holub, Petr Džubák, Marián Hajdúch

**Affiliations:** ^1^ Institute of Molecular and Clinical Pathology and Medical Genetics, University Hospital Ostrava, Ostrava, Czechia; ^2^ Institute of Molecular and Clinical Pathology and Medical Genetics, Faculty of Medicine, University of Ostrava, Ostrava, Czechia; ^3^ Department of Pathology, EUC Laboratoře CGB a.s., Ostrava, Czechia; ^4^ Department of Surgery, University Hospital Ostrava, Ostrava, Czechia; ^5^ Department of Surgical Studies, Faculty of Medicine, University of Ostrava, Ostrava, Czechia; ^6^ Department of Pathology, Znojmo Hospital, Znojmo, Czechia; ^7^ The Fingerland Department of Pathology, Charles University, Faculty of Medicine in Hradec Králové, Hradec Králové, Czechia; ^8^ The Fingerland Department of Pathology, Charles University, University Hospital Hradec Králové, Hradec Králové, Czechia; ^9^ Department of Clinical and Molecular Pathology, University Hospital Olomouc, Olomouc, Czechia; ^10^ Department of Clinical and Molecular Pathology, Faculty of Medicine and Dentistry, Palacký University, Olomouc, Czechia; ^11^ Department of Hematooncology, University Hospital Ostrava, Ostrava, Czechia; ^12^ Department of Hematooncology, Faculty of Medicine, University of Ostrava, Ostrava, Czechia; ^13^ Institute for Molecular and Translational Medicine, Faculty of Medicine and Dentistry, Palacky University Olomouc, Olomouc, Czechia

**Keywords:** amyloidosis, nodular pulmonary amyloidosis, amyloidoma, AH amyloidosis, pneumothorax

## Abstract

Amyloidosis is a rare metabolic disorder primarily brought on by misfolding of an autologous protein, which causes its local or systemic deposition in an aberrant fibrillar form. It is quite rare for pulmonary tissue to be impacted by amyloidosis; of the three forms it can take when involving pulmonary tissue, nodular pulmonary amyloidosis is the most uncommon. Nodular pulmonary amyloidosis rarely induces clinical symptoms, and most often, it is discovered accidentally during an autopsy or *via* imaging techniques. Only one case of nodular pulmonary amyloidosis, which manifested as a spontaneous pneumothorax, was found in the literature. In terms of more precise subtyping, nodular amyloidosis is typically AL or mixed AL/AH type. No publications on AH-dominant type of nodular amyloidosis were found in the literature. We present a case of an 81 years-old male with nodular pulmonary AH-dominant type amyloidosis who presented with spontaneous pneumothorax. For a deeper understanding of the subject, this study also provides a review of the literature on cases with nodular pulmonary amyloidosis in relation to precise amyloid fibril subtyping. Since it is often a difficult process, accurate amyloid type identification is rarely accomplished. However, this information is very helpful for identifying the underlying disease process (if any) and outlining the subsequent diagnostic and treatment steps. Even so, it is crucial to be aware of this unit and make sure it is taken into consideration when making a differential diagnosis of pulmonary lesions.

## Introduction

Amyloidosis is a rare metabolic disease caused primarily by misfolding of an autologous protein, which results in its local or systemic deposition in an abnormal fibrillar form. Amyloidosis can be further classified as localised or systemic, hereditary or acquired, and according to the type of protein (more precisely, the fibril type) that leads to the manifestation of the disease. The characteristics of the fibril type provide the most accurate classification of amyloidosis. In general, the AA type is the most common type of amyloidosis, characterised by the presence of the serum amyloid A protein (one of the acute phase proteins) as the primary component. AL amyloidosis, which is composed of light immunoglobulin chains, ranks second in prevalence (it should be, however, noted that this type is the one most often encountered in clinical practice). ATTR, beta-2, and AH-dominant amyloidosis are other, much less common, subtypes (the precursors in the given case include various forms of transthyretin, beta-2 microglobulin and heavy immunoglobulin chains, respectively) [1–4].

**FIGURE 1:**
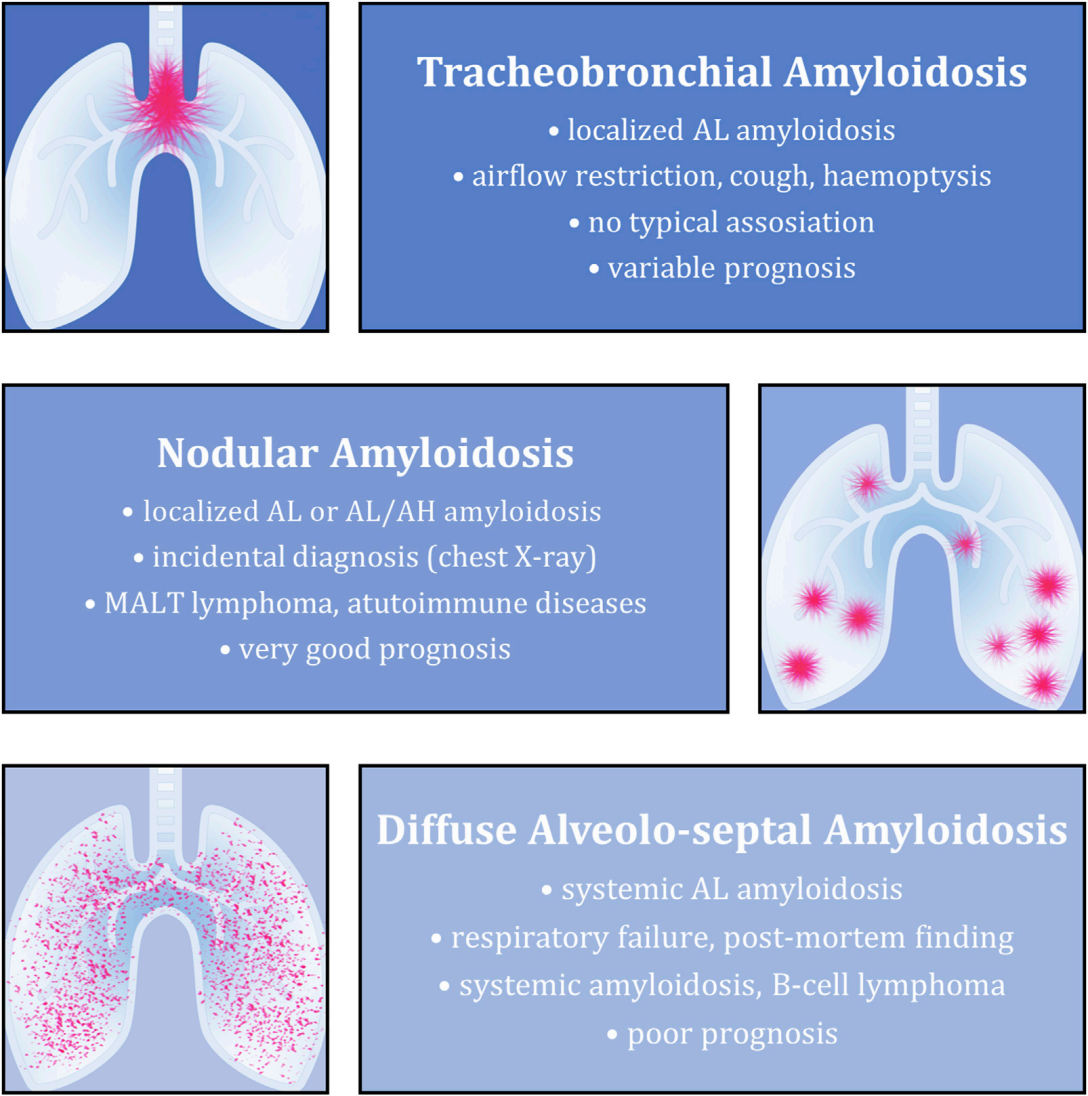
Overview of the most typical features of individual types of amyloidosis with pulmonary involvement. The described aspects (from top to bottom) include the most common associated amyloid type, clinical picture (if present), underlying diseases and final prognosis.

It is quite uncommon for pulmonary tissue to be affected by amyloidosis, especially when discussing a local form. Histopathologically, pulmonary involvement typically takes one of three forms—tracheobronchial, nodular, or diffuse alveolo-septal amyloidosis (Figure 1). The nodular form is the most uncommon in this group. It rarely causes clinical symptoms, and is mostly revealed as an accidental finding detected during imaging investigation for a different reason or at autopsy. This subtype has the best prognosis [1, 5, 6].

In terms of more precise subtyping, nodular amyloidosis is typically AL or mixed AL/AH type. Although it is typically a localised form of amyloidosis, pulmonary manifestation of systemic AA form can occur in a minority of cases. Accurate subtyping is extremely beneficial because it can aid in determining the causative disease (if present) and selecting the appropriate treatment [1, 2, 7–9].

Only one case of nodular pulmonary amyloidosis, which manifested as a spontaneous pneumothorax, was found in the literature [10], but it was not well described, and the exact correlation with amyloidosis was not established. Furthermore, no publication describing a case of a AH-dominant type (i.e., Ig heavy-chain–associated) nodular amyloidosis was discovered during the literature search.

We would like to present a case of a patient with a nodular pulmonary AH-dominant type amyloidosis who presented with spontaneous pneumothorax. This paper also includes a literature review on cases of nodular pulmonary amyloidosis where accurate amyloid fibril subtyping was performed to provide a more thorough understanding of the issue. The main goal of this article is to emphasise that although the given lung involvement is quite uncommon, it should still be considered when making a differential diagnosis. Additionally, precise amyloid subtyping can aid in determining the overall strategy for diagnosis and treatment. The case report itself was created using the CARE checklist (Supplementary Material S1).

## Case description

We report the case of an 81 years-old man who was hospitalized in a district hospital a spontaneous left-sided pneumothorax. Bronchoscopy was performed and chest drain inserted; however, as the patient’s condition was not improving, he was transferred to the University Hospital Ostrava for further (surgical) treatment.

On admission, the patient’s general condition was stable. The patient was fully oriented and cooperative. Physical examination revealed left-sided subcutaneous emphysema and an air leak from the chest drain incision. Arterial pressure was 120/80 mmHg, heart rate was 78 beats per minute, and respiration rate was 20 per minute, all of which were within normal limits. Other findings were not clinically significant.

The patient’s personal history included the following diagnoses—bronchial asthma, TIA/CVA (transient ischemic attack, cerebrovascular accident) from the right internal carotid artery basin (with left-sided hemiparesis), vertebrogenic algic syndrome of the thoracic and lumbar spine, benign prostatic hyperplasia, cataract surgery, total right hip replacement and Th11 (11th thoracic vertebra) fracture. He denied having any allergies. The medical family history was unremarkable. Before developing pneumothorax, however, the patient’s condition was good and appropriate to his age and he suffered from no new medical conditions.

After admission to the University Hospital Ostrava, a biportal thoracoscopic revision was performed. The camera port was placed between the mid- and anterior axillary lines in the 7th intercostal space, and the working incision was placed near the anterior axillary line in the 4th intercostal space. Multiple emphysematous bullae with scarred lung parenchyma were found in the apical part of the upper lobe of the left lung. A 3 × 3 cm tumour with perforation of the surrounding lung parenchyma was also found at the edge of this lobe, causing the pneumothorax. A non-anatomical stapled wedge resection of the tumour and all bullous lung tissue was performed. Outside the mentioned area, the lung tissue showed no signs of other pathology.

**FIGURE 2:**
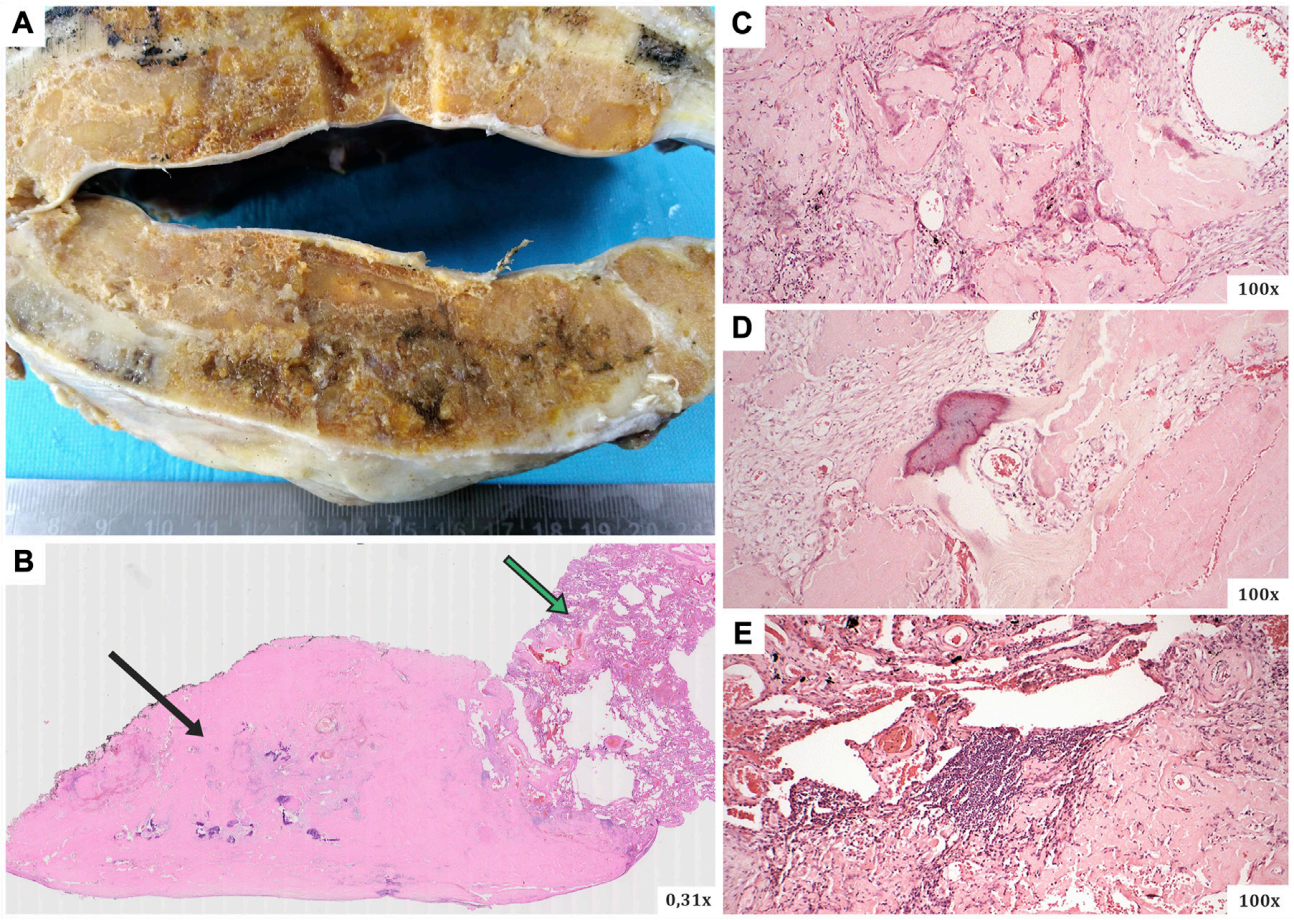
**(A)** The macroscopic appearance of the surgically resected lesion (a smooth flattened tumour of a whitish colour and solid consistency), **(B–E)** microscopic views of slides in haematoxylin-eosin staining. Microscopically, cross-sections of the lung tissue with a defined nodular focus produced by an amorphous eosinophilic matrix (typical for the morphology of the amyloid deposits). *Black arrow*–the main part of the amyloidoma mass (with numerous calcifications), *green arrow*–residual lung tissue.

**FIGURE 3:**
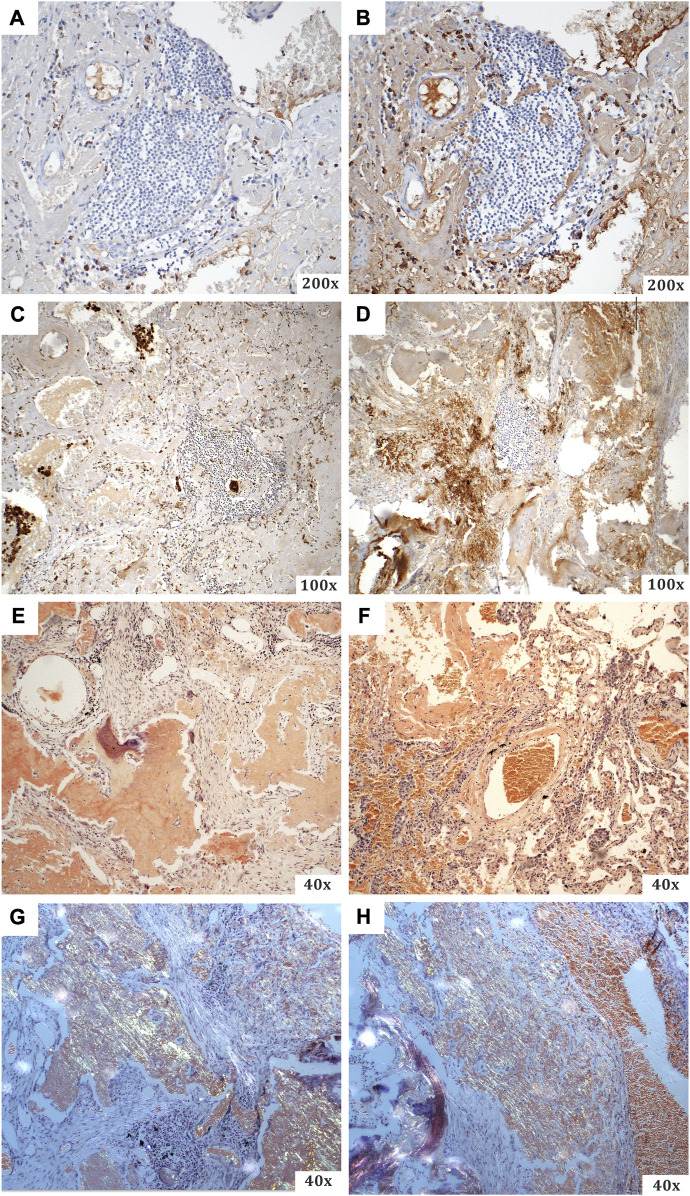
A summary of the special staining techniques used during the histopathological evaluation. **(A)** CD68 (with visible B-lymphocytes of plasmacytoid differentiation), **(B)** CD138 (from the same specimen area as the previous one), **(C)** kappa light chains, **(D)** lambda light chains, **(E–H)** Congo red [**(E,F)** light microscopy; **(G,H)** polarised light imaging with typical apple green dichroism].

The resected material was sent to the Department of Pathology, where a comprehensive histopathological, immunohistochemical, immunofluorescence and proteomic examination was performed. Macroscopically, a smooth flattened tumour of a whitish colour and solid consistency was visible, 2.8 × 2.3 cm in size. Cross-sections of the lung tissue with a demarcated nodular focus formed by an amorphous eosinophilic matrix, consistent with the amyloid appearance, were observed microscopically (Figure 2). Histochemical evidence revealed positivity in Congo red and Saturn red staining. These structures exhibited typical dichroism (apple green) in polarised light. Numerous multinucleated CD68-positive histiocytes were present around the lesion. CD79a and CD138 positive B-lymphocytes of plasmacytoid differentiation were also dispersed in the lung tissue. Selective immunohistochemical staining was used to detect lambda and kappa immunoglobulin light chains, but only polytypic expression was observed (i.e., no restriction; Figure 3). Areas of dystrophic calcification and metaplastic ossification were also present. Other findings, apart from a large number of subserous emphysematous bullae and pigmentophages, were insignificant. Immunofluorescence analysis revealed no positivity for the AA, TTR, or immunoglobulin light chains. The likelihood of an AL amyloidosis diagnosis was, therefore, extremely low.

Following that, a proteome analysis was performed using the LMD-LC/MS (thermal film-based laser microdissection and liquid chromatography coupled with mass spectrometry). The peptides were isolated from the Congo red positive sections of the FFPE (formalin-fixed and paraffin-embedded) lung tissue specimens, which were subsequently laser microdissected. Proteins were solubilized and digested to peptides for shotgun proteomics. Liquid chromatography was used to separate peptides, and mass spectrometry was used for examining individual peptides. The MaxQuant search engine was used to identify and quantify the acquired data. As a result, the relative intensity of each amyloid-forming protein was calculated as a percentage of the total intensity of all amyloid-forming proteins. The amyloid subtype AH was assigned to the amyloid-forming protein Ig alpha-1 chain C region, which had the highest relative intensity (60.4%). Other amyloid-forming proteins were also discovered; their relative proportion was, however, much lower—to be specific, these low-represented amyloid proteins included Ig gamma-1 chain C area (7.2%), Ig kappa chain C region (6.0%), Immunoglobulin lambda-like polypeptide (6.0%), and Ig kappa chain V-III region B6 (3.1%). The remaining proteins included Serum Amyloid P, Apolipoprotein E, Apolipoprotein A-IV, and others. The obvious preponderance of Ig heavy chains led to the diagnosis of primary nodular pulmonary amyloidosis of the AH-dominant type was established.

The patient was observed postoperatively in the ICU (intensive care unit) of the surgical department. No complications occurred—the patient was lucid, afebrile, compensated, he felt well, the wound healed per primam, with adequate amount of discharge from the chest drain, the air leak dwindled until it finally disappeared. In stable condition, he was then transferred to the standard department of the surgical clinic, where his condition continued to improve. He was finally discharged to outpatient care on postoperative Day 7. On follow-up at 2 months, the patient was completely well, and the lung X-ray was free of pathology.

Due to the ambiguous results of the histopathological examination, further investigations to rule out the presence of haematological malignancy was performed. Two months later, the patient underwent a thorough examination at the Department of Haematooncology. Bone marrow aspiration from the right iliac crest yielded no pathological findings on cytology, flow-cytometry and cytogenetics. Additional blood tests detected no evidence of monoclonal gammopathy with negative serum and urine immunofixation and normal free light chains ratio. The blood count was normal (leukocytes 5.8 × 10^9^/L, haemoglobin 135 g/L, platelets 367 × 10^9^/L), with 9% lymphocytes and 82% neutrophils. Beta 2 microglobulin was slightly elevated with a value of 2.96 mg/L, lactate dehydrogenase was normal (2.44 ukat/L). Monoclonal gammopathy was not detected, serum and urine immunofixation were negative, and the free light chains ratio (FLCr) was normal, without an increase in free light chains. Other biochemical tests were also normal. Finally, abdominal fat aspiration was performed with no evidence of amyloid presence. Haematological examination excluded plasma cell dyscrasias (monoclonal gammopathy, AL amyloidosis) because of negative findings of the M protein, light chains, clonal plasma cells in the bone marrow and subcutaneous fat infiltration.

Unfortunately, the subsequent progress of the patient is unknown as any attempts to contact him were unsuccessful.

## Review of the literature

Using the keywords “amyloidoma,” “nodular amyloidosis,” “lung,” “pulmonary,” “pneumothorax” and their combination, a total of 654 articles were found in the PubMed database. The search criteria were full-text publications available from 1960 to the present (“all time” search). Following a thorough review, individual article abstracts that were irrelevant to the topic, as well as duplicates, were excluded. As a result, 90 papers on nodular pulmonary amyloidosis cases were included in the next stage of the analysis. During further processing, 70 articles were discarded because of the missing histopathological verification or precise molecular subtyping of amyloid protein. Finally, 20 case reports were included in the final analysis, as shown in Table 1 (which also includes the presented patient’s case).

**TABLE 1:** An overview of cases of nodular pulmonary amyloidosis with a precisely determined type of amyloid protein reported since the year 1960.

Case	Year	Country	Age/Sex	Initial presentation	Underlying condition	Location	Number of nodules	Maximum size	Amyloid fibrils type	Outcome	References
1	2023	Czechia	81/M	Spontaneous pneumothorax	None	left upper lobe	Solitary	28 mm	Heavy chain	? months: no progression	current case
2	2022	Japan	67/F	None	Sjögren’s syndrome	bilateral, lower lobes	Multiple	6 mm	Light chain, kappa	60 months: no progression	[8]
3	2013	Poland	67/F	Resting dyspnoea, malaise, weight loss	MGUS	bilateral	Multiple	—	Transthyretin	3 months: progression, death	[4]
4	2012	Taiwan	77/F	Productive cough, intermittent fever, shortness of breath	Waldenström’s macroglobulinemia	lingual lobe	Solitary	32 mm	Light chain, lambda	—	[15]
5	2012	Japan	65/M	Weight loss	None	Right middle lobe	Solitary	—	Light chain, lambda	? months: no progression	[14]
6	2011	Italy	63/M	Shortness of breath, cough	None	Right lower lobe	Solitary	27 mm	Light chain, kappa	24 months: regression	[13]
7	2010	India	41/F	Non-productive cough, dyspnoea	Sjögren’s syndrome	Bilateral, lower lobes	Multiple	—	light chain, lambda	24 months: no progression	[3]
8	2010	Japan	62/M	Dry cough	None	Right lung	Multiple	30 mm	serum amyloid protein A	6 months: regression	[16]
9	2009	United States	58/F	None	Sjögren’s syndrome, MCTD (CREST type)	Bilateral	Multiple	—	predominantly beta-2 microglobulin	—	[2]
10	2009	United States	82/M	Left pleural effusions	None	Left lower lobe	Solitary	—	Transthyretin	—	[7]
11	2007	Spain	67/F	None	Rheumatoid arthritis	Bilateral	Multiple	—	Serum amyloid protein A	—	[12]
12	2005	Israel	69/M	Long-lasting pneumonia	Lymphoplasmacytoid lymphoma	Left upper lobe	Solitary	210 mm	Light chain, lambda; heavy chain, IgG	—	[17]
13	2005	Japan	59/F	Obstinate cough	Sjögren’s syndrome, HAM/TSP	Bilateral	Multiple	—	Light chain, kappa	108 months: death	[18]
14	2001	Germany	63/F	Swollen right upper eyelid	MALT lymphoma	Bilateral	Multiple	—	Light chain, lambda	18 months: regression	[19]
15	1999	Japan	72/M	Productive cough	None	Right lower fields	Multiple	30 mm	Light chain, kappa	24 months: regression	[5]
16	1997	Japan	63/F	None	None	Bilateral, upper fields	Multiple	30 mm	Light chain, lambda	60 months: death	[11]
17	1993	United Kingdom	75/F	Diarrhoea, weight loss	Crohn’s disease	Bilateral, lower lobes	Multiple	30 mm	Serum amyloid A protein	3 days: death	[20]
18	1991	United Kingdom	56/M	Pleural pain, haemoptysis	SCLC	Bilateral	Multiple	10 mm	Light chain, kappa	? months: regression	[6]
19	1989	Japan	77/M	None	None	Right lower lobe	Multiple	—	Light chain, lambda	? months: regression	[21]
20	1984	United States	86/F	Weakness, weight loss	None	Bilateral	Multiple	—	Light chain, kappa and lambda	36 months: death	[22]
21	1968	Japan	53/F	Cough, exertional dyspnoea	Sjögren’s syndrome	Left lung	Multiple	20 mm	Light chain, kappa	? months: no progression	[23]

CREST, calcinosis, Raynaud phenomenon, oesophageal dysmotility, sclerodactyly and telangiectasia; F, female; HAM/TSP, human T-lymphotropic virus-associated myelopathy/tropical spastic paraparesis; M, male; MALT, mucosa-associated lymphatic tissue; MCTD, mixed connective tissue disease; MGUS, monoclonal gammopathy of undetermined significance; SCLC, small cell lung cancer.

As the description of the search shows, determining the exact type of amyloid fibrils is only performed in a minority of cases. The authors are usually satisfied with the fact that the finding excludes AA amyloid and, hence, AL amyloid is assumed based on an underlying disease.

A closer look at the reported cases selected for further analysis reveals that the majority of patients (57%) are women. The mean age is 66.8 years (range 41–86 years, median age 67 years). A quarter of the patients had no clinical symptoms at the time of diagnosis, the findings were incidental (either on imaging methods or at autopsy). Twelve patients (57%) showed various pulmonary symptoms (from banal cough to spontaneous pneumothorax in our case). No underlying co-morbidity was present in nine patients (43%). Of the remaining 57% who had a primary underlying illness, Sjögren’s syndrome (a total of five cases—24%) was the most common. As for the nature of the pulmonary impairment, it was most often multiple nodules (71%) that were located bilaterally (73.3% of all cases with multiple nodules).

According to the molecular subtyping of amyloid fibrils, AL amyloidosis was the most common type in the published reports (a total of 13 cases—62%). The distribution of the types of light immunoglobulin chains was more or less uniform—six cases of kappa and lambda each, a mixed involvement in one case. Of the remaining types of amyloidosis, serum A protein was detected in three cases (14%), transthyretin in two (10%), beta-2 microglobulin (5%) and heavy immunoglobulin chains in one patient each. A case of combined AL/AH involvement was reported as well.

Additionally, it is also necessary to mention the study by Grogg et al. [9], which presents a thorough examination of clinicopathological data from 18 patients with nodular amyloidosis. Unfortunately, as this study did not report detailed information about individual patients, we could not include it into the table of cases (Table 1). This study, however, revealed interesting results. In particular, mass spectrometry examination of the amyloid deposits revealed immunoglobulin light chains with a majority of kappa (77%) in all 18 cases. This differed from 14 patients with systemic form AL amyloidosis, which was characterised with a predominance of light chains of the lambda type. Moreover, 13 out of the 18 patients with nodular pulmonary amyloidosis showed immunoglobin heavy chain co-deposition, indicating mixed AL/AH type amyloidosis. This was another feature distinguishing it from the systemic AL type. The study authors also note that there is an important association between the development of nodular pulmonary amyloidosis and the presence of an underlying lymphoplasmacytic neoplasm, which should be considered when deciding on the next course of action in patients with similar findings.

## Discussion

As stated in the introduction, pulmonary amyloidosis is a rare diagnostic entity that should not be overlooked in the differential diagnosis of lung lesions. Some units, particularly diffuse alveolo-septal or tracheobronchial amyloidosis, can directly threaten the patient’s life and frequently manifest in a wide range of symptoms. These particularly include significant respiratory symptoms such as a severe cough, haemoptysis, dyspnoea, shortness of breath or even the gradual development of respiratory failure (especially when diffuse damage is present) [1, 3, 8]. Nodular pulmonary amyloidosis, on the other hand, is an indolent disease. Associated symptoms are relatively rare, mild, and non-specific, although more severe manifestations, such as haemoptysis or even pneumothorax, can occur at times (see Table 1). This type of pulmonary amyloidosis has the best prognosis of the entire group of described diseases [1, 5, 7, 11].

In most cases, the detection of typical regular round lesions in nodular pulmonary amyloidosis is an accidental finding of (particularly) imaging methods (mostly chest radiography) when the lungs are being examined for a different reason. It is also frequently discovered by chance during an autopsy [8, 12, 13]. Since pulmonary amyloidosis cannot be distinguished solely by the appearance of nodules provided by imaging techniques, a biopsy is required to accurately determine the pathological changes. The main question is whether or not the focus is a malignant tumour with a similar morphological profile. This is a critical piece of information since the patient’s subsequent treatment process and prognosis are completely different. In most cases, imaging-guided fine needle aspiration biopsy, a very gentle technique, is sufficient. However, if this is not possible, thoracotomy is performed to obtain a representative sample [2, 14]. Alternatively, the disease can be detected as a secondary finding during lung surgery performed for another reason (as in our case).

The histopathological analysis of the taken sample is the most important examination in determining the diagnosis of amyloidosis. The detection of apple green birefringence in polarised light after Congo red staining is the most notable and typical finding. Further analysis is performed, primarily using immunohistochemical analyses, to accurately determine the type of amyloidosis (based on the composition of protein fibrils) [4, 12, 13]. The AL type is the most common finding in the nodular pulmonary form of amyloidosis. It can be distinguished from the other types by the accumulation of monoclonal immunoglobulin light chains (lambda or kappa, with kappa typically prevailing). The mixed AL/AH type, which is characterised by the deposition of both light and heavy chains of immunoglobulins, is also fairly prevalent. These types are frequently linked to lymphoproliferative diseases, mainly lymphoplasmacytic B-cell neoplasms in the spectrum of MALT (mucosa-associated lymphoid tissue) lymphoma. The AA type is significantly less common and is distinguished by the deposition of serum amyloid protein A. It is typically linked to systemic inflammatory processes. Other subtypes, including AH-dominant, are extremely rare, as has been stated numerous times [1–4, 9, 13]. It was an AH-dominant type in our case, with heavy monoclonal immunoglobulin chains accumulating. As with AL amyloidosis, it is most frequently associated with lymphoproliferative diseases, particularly plasma-cell neoplasms.

An accurate identification of the type of amyloid is rarely established, as it is often a complicated process. This is supported by our findings, which show that the exact type of amyloid was described only in 20 out of the 90 case reports. However, this information is extremely useful, particularly in determining the underlying pathological process (if present) and defining the subsequent diagnostic and therapeutic procedures. The treatment varies from type to type, but in general, the nodular pulmonary amyloidosis itself, if not removed during the diagnostic process, does not require any intervention. However, regular observation is necessary. Even in the absence of a predisposing disease, chemotherapy is recommended to suppress plasma cell dyscrasia in the systemic form of AL type (which should also apply to AL/AH and AH types) [1, 3, 7, 12].

In our case, the disease was discovered by chance during the surgical revision of spontaneous pneumothorax (which is also a rare manifestation). Although the patient was of AH-dominant type suggests the presence of a haemato-oncological disease, no such pathology was discovered during specialised examinations. As a result, we can assume that regular observation will be completely sufficient in our patient’s case and that no further complications will arise because the pathological focus was surgically removed.

## Conclusion

Our case demonstrates that, despite its rarity, nodular pulmonary amyloidosis can lead to a serious health condition, such as spontaneous pneumothorax. The precise amyloid subtyping can aid in determining the overall strategy for diagnosis and therapy. Unfortunately, despite the precise determination of the type, it is not always possible to identify the primary pathology that caused the occurrence of amyloidosis. Even so, it is important to be aware of this nosological entity and not to overlook it when conducting a differential diagnosis of lung pathologies.

## Patient perspective

It was not possible to retrieve it as the patient could not be contacted after the treatment and unfortunately, we do not know about his further fate.

## Data Availability

The original contributions presented in the study are included in the article/Supplementary Material, further inquiries can be directed to the corresponding author.
