# Supplementary figures and images for: A unique case of AH-dominant type nodular pulmonary amyloidosis presenting as a spontaneous pneumothorax: a case report and review of the literature
Source: Pathol Oncol Res. 2023 Sep 22;29:1611390. doi: 10.3389/pore.2023.1611390 (PMC10556250; doi:10.3389/pore.2023.1611390)

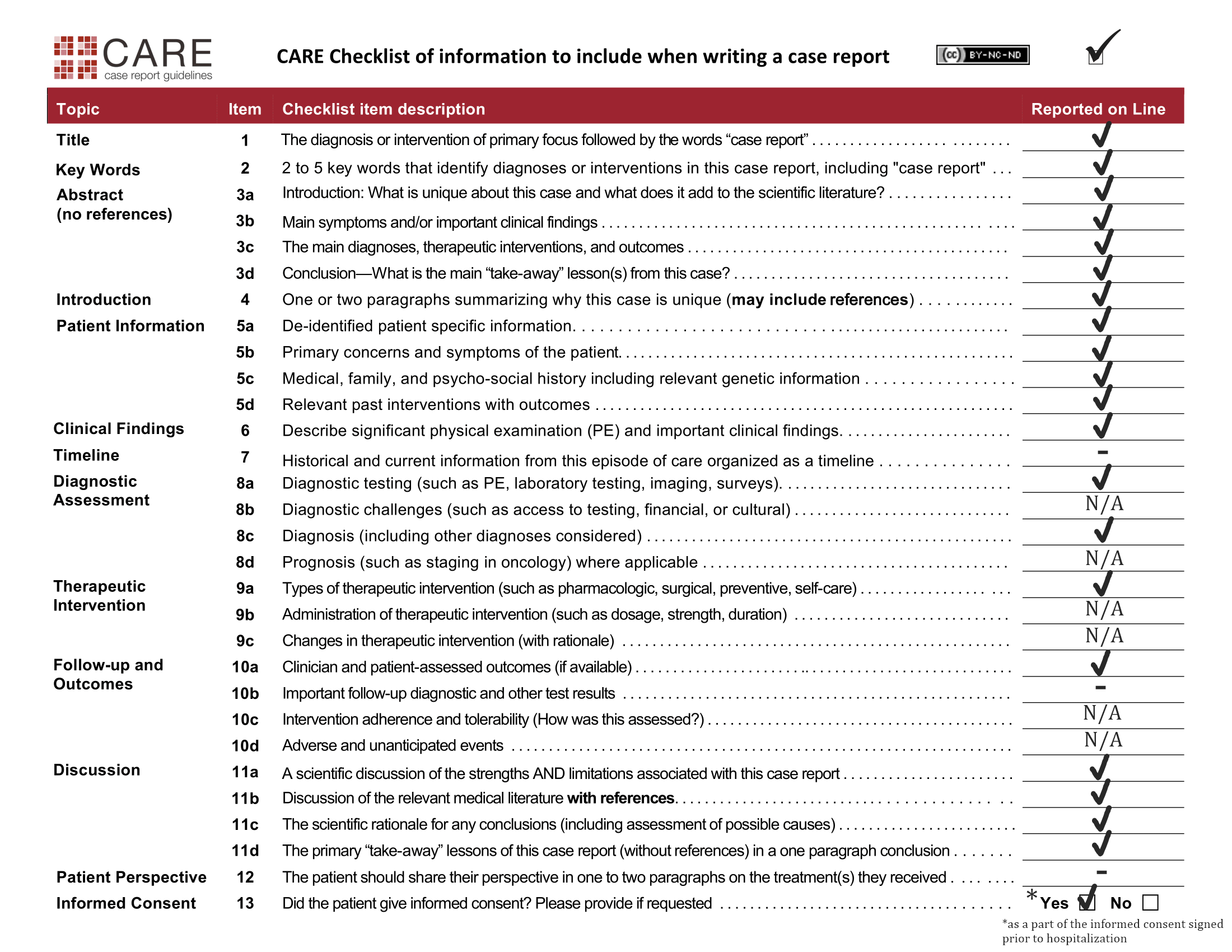

Supplement: Supplementary file 1 [file Image1.png]
